# Never too late? Quadruplets at the age of 65 years

**DOI:** 10.1007/s00404-021-06127-2

**Published:** 2021-06-19

**Authors:** Larry Hinkson, Christof Dame, Thorsten Braun, Irit Nachtigall, Wolfgang Henrich

**Affiliations:** 1 Department of Obstetrics, Charité-Universitätsmedizin Berlin, 1335, 10117 Berlin, Germany; 2 Department of Neonatology, Charité-Universitätsmedizin Berlin, 1335, 10117 Berlin, Germany; 3 Department of Preventive Medicine and Hygiene, HELIOS Hospital Bad Saarow, Bad Saarow, Germany; 4 Department of Anesthesiology and Operative Intensive Care Medicine (CCM, CVK), Charité-Universitätsmedizin Berlin, Berlin, Germany

**Keywords:** Advanced reproductive age, Cross-border reproductive care, In vitro fertilization, Quadruplets, Case report

## Abstract

**Background:**

We discuss the challenges of multiple pregnancy at very advanced reproductive age.

**Case presentation:**

We present the case of a quadruplet pregnancy at the maternal age of 65 following *in-vitro* fertilization (IVF) with donor eggs and sperm, involving cross-border reproductive care. All children born were at 25 weeks’ gestation and survived; however, poor neurodevelopmental outcome remains a major concern in one child.

**Conclusions:**

The use of reproductive technology to achieve a multiple pregnancy at such an advanced post-menopausal age generated a debate on ethical, psychosocial and medical questions. We share this debate and highlight the need to reconsider international guidelines for women of advanced reproductive age.

## Background

News media houses compete to report headlines on cases of women of advanced reproductive age. Quadruplets born from a mother at 65 years of age, however, indicate an extreme on the developments in reproduction medicine. Over the past years, advanced reproductive age (> 45 years or in the postmenopausal period) is gaining more impact in assisted reproductive technology [1, 2]. These pregnancies require experienced interdisciplinary care (Obstetrics, Cardiology, Anesthesiology, Neonatology, Psychiatry), but cases such as reported here go far beyond the current recommendations such as that of the Ethics Committee of the American Society for Reproductive Care [2]. Furthermore, our case sheds light on significant cross-border reproductive care within Europe, where extreme contrary positions of restrictive and liberal regulations exist [3, 4].

## Case presentation

A native German (Gravida 17, Para 13) had 13 children, the youngest (also after IVF) being 10 years of age. At the age of 64 years, she became pregnant following transfer of four embryos in the Ukraine. After returning to Germany, the first trimester embryosonography showed a quadruchorionic pregnancy without abnormalities. Intensive counseling about the specific risk factors for maternal health and prematurity at the limits of viability was provided. The patient declined unselected embryo reduction. A prophylactic cervical cerclage was performed at 13 weeks. Ultrasound examination at 22 weeks showed moderate growth discrepancy between fetuses, but normal fetal and maternal Doppler flows. Maternal echocardiography revealed slight, left atrial dilatation due to mild mitral valve prolapse. At 25 weeks + 4 days of gestation, the patient developed arterial hypertension and shortness of breath. She refused hospital admission, treatment of arterial hypertension, and antenatal corticosteroids for fetal lung maturation. Ten hours later, the patient returned with preterm labor. Betamethasone (12 mg) was given for fetal lung maturation. Tocolysis with indomethacin (100 mg) was commenced, but shortly thereafter, urgent caesarean section was required due to contractions and maternal dyspnea. Peridural anesthesia was performed to avoid a sudden drop in blood pressure. Radial arterial access was also established, and phenylephrine was given. The deliveries were uneventful, including late cord clamping, to reduce the risk of neonatal intraventricular hemorrhage (IVH). Prophylaxis against uterine atony and postpartum hemorrhage with a prostaglandin infusion (sulprostone 50 µg/h) and 2 g tranexamic acid were administered and total blood loss was 800 ml. The hemoglobin concentration dropped to 9.4 g/dl, but remained stable. Fresh frozen plasma was given. Mild maternal pulmonary edema, required end-expiratory pressure ventilation via high flow CPAP with oxygen supplementation (max. FiO_2_ 0.3) for 44 h, without vasoactive medication. A temporary hyperthermia, associated with a mild systemic inflammatory response syndrome, normalized before transfer from the intensive care unit 48 h after delivery. After mobilization, the mother began “kangarooing” with the quadruplets. Breast-milk production was achieved by manual stimulation and milk pumping.

One female (I) and three male (II–IV) extremely low birth weight (ELBW) infants were born at completed 25 weeks + 5 days of gestation. All four had birthweights appropriate for gestational age (I 655 g; II 960 g; III 680 g; IV 745 g; 19th to 72nd percentile). Respiratory distress syndrome required application of surfactant and invasive mechanical ventilation in the boys, but only nasal CPAP in the girl. Resuscitations were uncomplicated; all infants received prophylactic indomethacin to prevent IVH. All had elevated erythroblast counts, and two exhibited neutropenia at birth. In addition to mild broncho-pulmonary dysplasia, two babies developed major complications: On day 3, baby II was diagnosed with IVH grade 3 + /2, with subsequent posthemorrhage hydrocephalus, requiring a ventriculo-peritoneal shunt later on. Shortly after the IVH, he developed hyponatremia and ascites, first suggesting inappropriate antidiuretic hormone secretion. Respiratory insufficiency with pulmonary hypertension (treated with inhaled nitric oxide) required drainage of the ascites, which was diagnosed as uroascites, resulting from rupture of the urinary bladder, possibly as complication of a urethral valve. After catheterization of the bladder and temporary renal insufficiency, the disorder was self-limiting without surgery. Baby (I) developed focal intestinal perforation on day 8, likely related to residual ductus omphaloentercius. Primary excision of the perforation without artificial anus was possible, and the baby developed well. All four infants were discharged on day 93 at 38 weeks + 6 days of corrected age. The mother was supported throughout the pregnancy and postnatally by our psychological support team and a far-reaching network for social-medical support was established.

Indeed, without the benefit of a highly skilled multidisciplinary team of obstetricians, midwives, cardiologists, internists, anesthetists, neonatologists and ongoing psychosocial support, the outcome of this pregnancy may have been faced with a higher risk of morbidity and mortality because of the unique challenges faced by extremely advanced maternal age and very early premature birth of quadruplets.

## Discussion and conclusion

### Challenges for the mother

Generally, the risk of early pregnancy loss is very high in quadruplet pregnancies (16.7%) [5]. Up to the limits of viability, this quadruplet pregnancy was surprisingly uneventful despite the advanced reproductive age. The patient declined questions on attempts to become pregnant after her pregnancy 10 years ago. Her unique situation may explain why consequences of prenatal counselling were refused, including fetal reduction. Earlier administration of antenatal corticosteroids for fetal (lung) maturation was also declined by the mother—one of few interventions with evidence for reducing mortality and morbidity of extremely immature infants [6].

Pregnancy in advanced age is a cardiovascular burden that can worsen any preexisting cardiac disorder resulting in life-threatening complications, particularly in multi-fetal or twin pregnancy [7–9]. High incidences of preeclampsia and diabetes warrant early screening [10–12].

When asked about her motivation to become pregnant at this age and to take tremendous risks for herself and for likely very preterm infants into account, the mother insistently referred to the wish of her youngest daughter to have one or more younger siblings. Since oxytocin release has been described as a “love and bonding” hormone, influencing neuroadaptation, sexual arousal and social bonding, it may play a role in the desire to become pregnant [13–15]. Thus, the question on the desire for oxytocin may deserve attention in the psychoneuroendocrinology research of postmenopausal pregnancy. The underlying hypothesis of addiction to oxytocin release is even more interesting if the semen donor is not the partner, and the pregnant postmenopausal woman acts as surrogate mother.

### Challenges for the infants

The individual risk for death or profound impairment of the infants appears to be higher due to advanced reproductive age, if one considers that mean carriage of quadruplets reaches approximately 28 weeks of gestation. But such associations cannot be confirmed in this report. According to the extremely preterm birth outcome data of the NICHD Neonatal Research Network, the expected survival rate individually calculated for each quadruplet ranged between 60 and 82% [16]. More recent data from Germany indicate a slightly higher survival rate for ELBW infants born at 25 completed weeks of gestation (84.2% in triplets and 79.5% in higher order multifetal pregnancies) [17]. All four infants survived; however, poor neurodevelopmental outcome remains the major concern.

The calculated individual survival rate, without moderate to severe neurodevelopmental impairment,^1^ ranged only between 13 and 22% [16]. If multifetal pregnancy is not considered as additional risk factor, higher survival rates without moderate to severe impairment (mean 44.5%) or without severe impairment (mean 61.4%), at 18–22 months of corrected age have been reported in ELBW infants born at 25 completed weeks of gestation [18]. More reliable are long-term outcome measures obtained at 5–6 years [19]. To date, at least the quadruplet baby with IVH suffers from long-term sequelae of extreme prematurity. We are not aware of valid data on the question how the very advanced reproductive age (> 65 years) might affect later requirements for individualized, neuro-developmental care and normal assessment in all four children.

Because this quadruplet pregnancy resulted from egg and semen donations, the risk of chromosomal abnormalities and structural anomalies may not be higher as in other cases of IVF and embryo transfer. However, the impact of epigenetic changes due to advanced reproductive age with higher risk of gestational complications such as hypertension, diabetes and hormone insufficiency is entirely unclear in humans and may deserve evaluation.

### Challenges for society

Early on in pregnancy, the case generated a debate on ethical, psycho-social and medical questions in our interdisciplinary team. This debate, however, focused on responsibility of the patient and responsibility of the doctors who initiated this pregnancy.

Notably, in Germany quadruplet pregnancy at the age of 65 years would not have been possible without cross-border reproduction medicine for several reasons; (a) IVF with donor eggs (but not donor semen) is prohibited in Germany, (b) eligibility for IVF is restricted to medical indications, couples and heterosexuals. However, IVF does not underlay legislative restriction regarding maternal age, while cost coverage by non-private health insurances for IVF is strictly age-limited (< 40 years in females, and < 50 years in males), and (c) according to national guidelines and as documented in the registry of the European Society of Human Reproduction and Embryology (ESHRE), transfer of four or more embryos is normally not practiced [20]. Prohibition of IVF with egg donation and restriction of eligibility for IVF are controversially discussed in the German society and legislative politics. Both issues result in cross-border reproductive care to Eastern (e.g. Ukraine, Czech Republic) or Southern (e.g. Spain) European countries [3, 21, 22].

While the mother argued on her fundamental human right in decision making on her reproductive activity independent of her age, there were questions raised on the implications for society with increasing late maternity in postmenopausal women and the financial burden in a system of public health coverage. Physicians also need to consider the aspects of beneficence, that is provision of benefit to the patient and nonmaleficence, the principle of doing no harm, when planning assisted reproduction in advanced menopausal women, especially in the light of the risks to the mother and children [23]. The protection of additional parties, including egg and semen donors, and importantly the children born with the highest risk for severe life-long disabilities were rather eclipsed into the background of the debate. Physicians in Europe need to be aware and adhere to the good practice guidelines of the European Society of Human Reproduction and Embryology (ESHRE) [21].

As treating physicians, we should not personally judge about moral, ethical, justice or beneficial issues relevant for this case. However, in our opinion, the human rights of the children need to be moved into the foreground of ongoing debates and considerations about medically assisted reproduction in our societies. Our report on the extreme case of quadruplets at maternal age 65 years, with anonymous egg and semen donations in cross-border reproduction medicine, confirms that the individual wish for having a child (across the natural limits of fecundity, in singles, lesbian and gay couples, transsexual people and any other alternate family arrangement) cannot be regulated by national (or even international) law or medical guidelines. Therefore, international bodies, such as the United Nations, urgently need to discuss intensively the social, legal and medical implications of the current and future practice in reproductive medicine and—most importantly—to finally strengthen the rights of children, such as information on their biological identity, born after anonymous egg and/or semen donation.

## Data Availability

Data sharing is not applicable to this article as no datasets were generated or analyzed during the current study.

## Abbreviations

IVF: In-vitro fertilization
IVH: Intraventricular hemorrhage
ELBW: Extremely low birth weight
ESHRE: European Society of Human Reproduction and Embryology

## Footnotes

1 Definition of severe neurodevelopmental impairment according to the criteria of NICHD Neonatal Research Network: Mental or Psychomotor Development Index ≤ 70, moderate or severe cerebral palsy, bilateral blindness, and/or bilateral hearing loss requiring hearing aids cochlear implants).
